# MYB Transcription Factors Regulate Glucosinolate Biosynthesis in Different Organs of Chinese Cabbage (*Brassica rapa* ssp. *pekinensis*)

**DOI:** 10.3390/molecules18078682

**Published:** 2013-07-22

**Authors:** Yeon Bok Kim, Xiaohua Li, Sun-Ju Kim, Haeng Hoon Kim, Jeongyeo Lee, HyeRan Kim, Sang Un Park

**Affiliations:** 1 Department of Crop Science, Chungnam National University, 99 Daehak-ro, Yuseong-gu, Daejeon 305-764, Korea; E-Mails: yeonbokkim@hanmail.net (Y.B.K.); lixiaohua2007@hotmail.com (X.H.L.); 2 Department of Bio-Environmental Chemistry, Chungnam National University, 99 Daehak-Ro, Yuseong-Gu, Daejeon 305-764, Korea; E-Mail: kimsunju@cnu.ac.kr; 3 Department of Well-being Resources, Sunchon National University, 413 Jungangno, Suncheon, Jeollanam-do, 540-742, Korea; E-Mail: cryohkim@sunchon.ac.kr; 4 Green Bio Research Center, Cabbage Genomics Assisted Breeding Supporting Center, Korea Research Institute of Bioscience and Biotechnology (KRIBB), Gwahangno 111, Daejeon 305-806, Korea; E-Mail: leejy@kribb.re.kr

**Keywords:** Chinese cabbage, gene expression, glucosinolate, transcription factor

## Abstract

In this study, we investigated the expression of seven MYB transcription factors (a total of 17 genes that included Dof1.1, IQD1-1, MYB28, MYB29, MYB34, MYB51, and MYB122 and their isoforms) involved in aliphatic and indolic glucosinolate (GSL) biosynthesis and analyzed the aliphatic and indolic GSL content in different organs of Chinese cabbage (*Brassica rapa*ssp. *Pekinensis*). *MYB28* and *MYB29* expression in the stem was dramatically different when compared with the levels in the other organs. *MYB34*, *MYB122*, *MYB51*, *Dof1.1*, and *IQD1-1* showed very low transcript levels among different organs. HPLC analysis showed that the glucosinolates (GSLs) consisted of five aliphatic GSLs (progoitrin, sinigrin, glucoalyssin, gluconapin, and glucobrassicanapin) and four indolic GSLs (4-hydroxyglucobrassicin, glucobrassicin, 4-methoxygluco-brassicin, and neoglucobrassicin). Aliphatic GSLs exhibited 63.3% of the total GSLs content, followed by aromatic GSL (19.0%), indolic GSLs (10%), and unknown GSLs (7.7%) in different organs of Chinese cabbage. The total GSL content of different parts (ranked in descending order) was as follows: seed > flower > young leaves > stem > root > old leaves. The relationship between GSLs accumulation and expression of GSLs biosynthesis MYB TFs genes in different organs may be helpful to understand the mechanism of MYB TFs regulating GSL biosynthesis in Chinese cabbage.

## 1. Introduction

**Figure 1:**
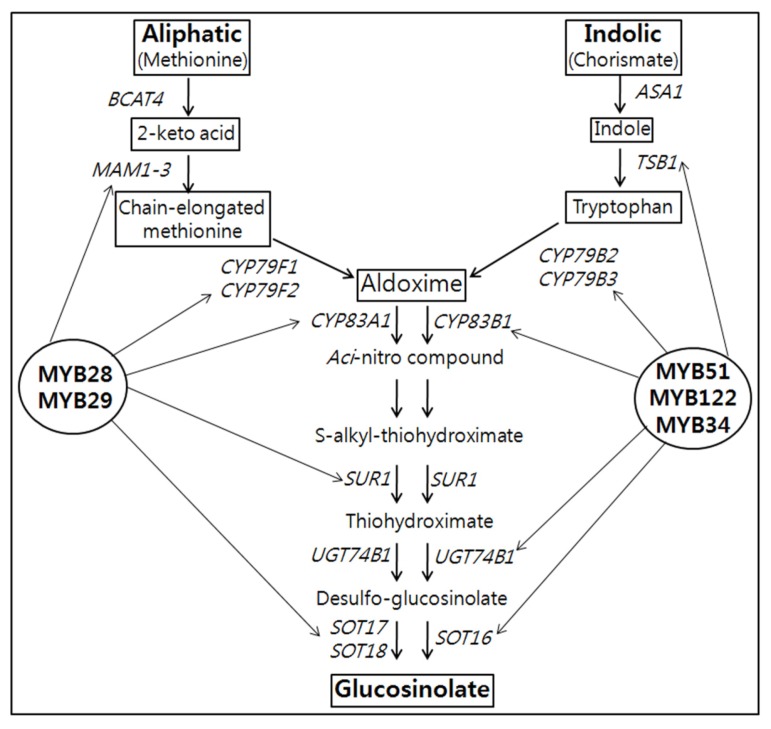
Schematic representation of the aliphatic and indolic glucosinolate biosynthesis in *B. rapa* [20]. The key MYB transcription factors involved in aliphatic and indolic glucosinolates are shown in bold. BCAT4, MAM1-3-Side-chain elongation; CYP79F1, CYP79F2, CYP79B2, CYP79B3, CYP83A1, CYP83B1, SUR1, UGT7B1-Core structure formation; ASA1, anthranilate synthase alpha 1; TSB1, tryptophan synthase beta 1.

Glucosinolates (GSLs), which are β-thioglucoside-*N*-hydroxysulfates (*cis*-*N*-hydroximinosulfate esters), are sulfur-rich anionic secondary metabolites that are derived from a glucose molecule and an amino acid. They occur as secondary metabolites of almost all plants of the order Brassicales (including the families Brassicaceae, Capparidaceae, and Caricaceae), and also in the genus *Drypetes* (family Euphorbiaceae) [1]. Approximately 200 different GSLs are known to occur naturally in plants [1,2]. GSLs play important roles in plant defense against herbivores and microbes [3] and have received considerable attention because of their anti-carcinogenic, anti-oxidative, and anti-microbial activities [4,5,6]. The amino acids methionine, tryptophan (Trp), and phenylalanine are the most prominent GSL biosynthetic precursors and are involved in the synthesis of aliphatic, indolic, and aromatic GSLs, respectively [3,7]. Indolic GSLs constitute an important group of Trp-derived secondary metabolites in brassicas where they function as defense compounds. Indolic GSLs and other GSLs are regulated by myrosinases, which convert them into biologically active nitrile, isothiocyanate, or thiocyanate forms [8]. During biotic and abiotic stress responses, GSL biosynthesis is regulated by a complex network of transcription factors (TFs) belonging to the R2R3-MYB family [9,10,11,12,13,14,15,16,17]. MYB28, MYB76, and MYB29 control the biosynthesis of high aliphatic GSLs [14,15,16,17], while MYB51, MYB122, and MYB34, alternatively called high indolic GSLs, can be manipulated to coordinately control the suite of enzymes that synthesize indolic GSLs (Figure 1) [9,12,13,18]. Skirycz *et al.* [12] reported that the DOF transcription factor AtDof1.1 (OBP2), which is inducible by herbivory and methyl jasmonate, is part of a regulatory network controlling GSL biosynthesis in *Arabidopsis*. 

*Arabidopsis* IQD1, a novel calmodulin-binding nuclear protein, stimulates GSL accumulation and plant defense [10]. The production of GSLs is intimately connected to primary sulfur metabolism. The final step of GSL core synthesis is the sulfation of the desulfo-GSL precursors [19]. Yatusevich *et al.* [20] showed that at least some of the ATPS (ATP sulfurylase), APK (APS kinase), and APR (APS reductase) genes are directly regulated by the R2R3-MYB TFs involved in the regulation of core GSL biosynthesis. Chinese cabbage (*Brassica rapa* ssp. *pekinensis*) belongs to the member of Brassicaceae family and is the most popular type of leafy vegetables widely used in East Asian cuisine. In Korean cuisine, it is the main ingredient of baechu Kimchi, the most common type of Kimchi. Although MYB TFs involved in GSL biosynthesis have been examined [9,10,11,12,13,14,15,16,17], gene expression studies on MYB TFs in different organs of Chinese cabbage have not been reported. In this study, we examined GSL accumulation and expression of R2R3-MYB TFs involved in the regulation of GSLs biosynthesis in different organs of Chinese cabbage. 

## 2. Results and Discussion

### 2.1. Identification of Transcription Factors Involved in GSL Biosynthesis

Eighteen TFs known to regulate GSL biosynthesis were identified in a *Brassica* database (http://brassicadb.org/brad/glucoGene.php) [21]. The open reading frame of MYB29-1 had 99% homology with MYB29-2 and was therefore excluded in this study. Wang *et al.* [21] identified 52 homologous *A. thaliana* GLS (AtGS) biosynthetic genes using BLASTN and BLASTP, on the basis of the draft *B. rapa* genome v1.0 and 41,174 annotated genes. *Brassica* TF genes were found to share almost 72%–92% nucleotide sequence identity with their *Arabidopsis* orthologs [21] (Table 1).

**Table 1:** Orthologs list of transcription factors involved in GSL biosynthesis in Chinese cabbage.

Group name	AGI	BrID	References
Dof1.1	At1g07640	Bra031588 (Dof1.1-1)	Skirycz *et al.* [12]
		Bra030696 (Dof1.1-2)	
IQD1-1	At3g09710	Bra034081 (IQD1-1-1)	Levy *et al.* [10]
		Bra001299 (IQD1-1-2)	
MYB28	At5g61420	Bra012961 (MYB28-1)	Gigolashvili *et al.* [14]; Hirai *et al.* [16]
		Bra035929 (MYB28-2)	
		Bra029311 (MYB28-3)	
MYB29	At5g07690	Bra005949 (MYB29)	Gigolashvili *et al.* [15]; Hirai *et al.* [16]
MYB34	At5g60890	Bra013000 (MYB34-1)	Celenza *et al.* [9]
		Bra035954 (MYB28-2)	
		Bra029350 (MYB28-3)	
		Bra029349 (MYB28-4)	
MYB51	At1g18570	Bra025666 (MYB51-1)	Gigolashvili *et al.* [13]
		Bra031035 (MYB51-2)	
		Bra016553 (MYB51-3)	
MYB122	At1g74080	Bra015939 (MYB122-1)	Gigolashvili *et al.* [13]
		Bra008131 (MYB122-2)	

**Figure 2:**
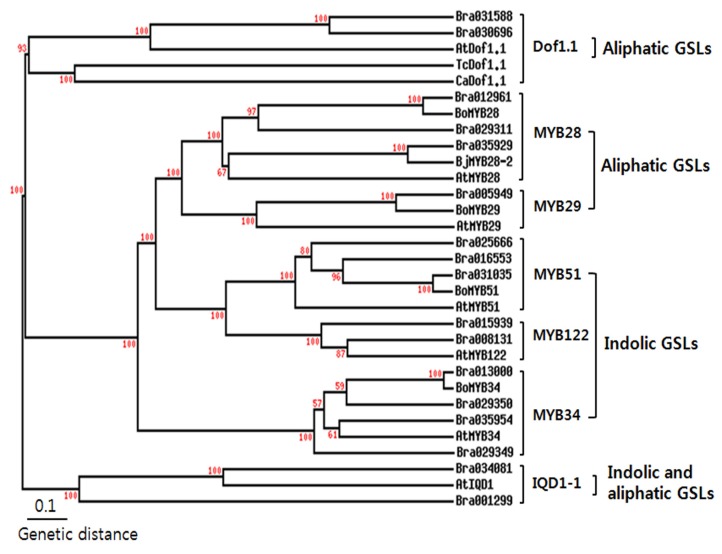
Phylogenetic tree of MYB transcription factors of *B. rapa* with other plants. The phylogenetic tree was generated by applying the cluster algorithm using TreeTop Phylogenetic tree. Bootstrap values were resample 100 times. GenBank Accession No. TcDof1.1 (*Theobroma cacao*, EOY34069), CaDof1.1 (*Cicer arietinum*, XP_004505871), BjMYB28-2 (*Brassica juncea*, AFX96282), BoMYB28 (*Brassica oleracea*, CBI71385), BoMYB29 (*Brassica oleracea*, BAM78212), BoMYB51 (*Brassica oleracea*, ACB59198).

*Arabidopsis* MYB51/HIG1 (high indolic glucosinolate 1) did not contain a typical nuclear localization signal (NLS) as revealed by PredictNLS [22]; however, an amino acid residue stretch KKRLIKK was detected that might act as a SV40-type NLS [14]. Software prediction of protein subcellular localization showed that all the transcription factors were present in the nucleus, except for Dof1.1-2, which according to the TargetP and ChloroP software programs, was placed in the chloroplasts (data not shown). Precursor amino acid synthesis and side chain elongation are known to take place in the chloroplasts [23,24]. The oxidation reactions of the GLS core pathway, catalyzed by CYP79 and CYP83 enzymes, take place at the ER-cytosol interface, as showed by the targeting of CYP7979F1- and CYPF2-reporter fusion proteins to the ER [25]. A phylogenetic tree was then constructed using the deduced transcription factor amino acid sequences of *A. thaliana* and *B. rapa* (Figure 2)*.* Wang *et al.* [21] reported that each transcription factor of *B. rapa* showed more than 70% sequence identity when compared to corresponding TFs in *A. thaliana*. As expected, MYB28 and MYB29-2, which are involved in aliphatic GSL production, were clustered together, whereas MYB51 and MYB122, which are involved in indolic GSL production, were clustered together. IQD1-1 and Dof1.1 were completely separate from the other TFs. 

### 2.2. Gene Expression of MYB TFs in Different Organs

**Figure 3:**
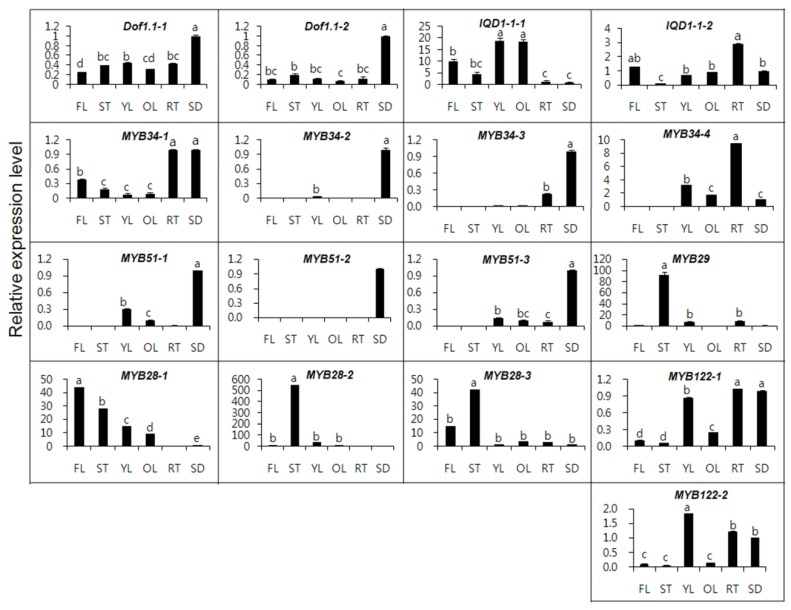
Expression of MYB transcription factors in different Chinese cabbage organs. FL-flower, ST-stem, YL-young leaf, OL-old leaf, RT-root, SD-seed. The longitudinal axis indicates the expression levels of genes relative to that of actin. Each value is the mean of three replicates, and error bars indicate SDs. Mean values indicated by the same letter are not significantly different at least significant differences (LSD) (*p =* 0.05).

Data regarding the transcription of MYB TFs in different organs are presented in Figure 3. *MYB28* and *MYB29*, involved in aliphatic GSLs showed extremely different patterns of expression among different organs, compared with other TFs. Gigolashvili *et al.* [14] reported that the R2R3-MYB TF MYB28/HAG1 (high aliphatic glucosinolate 1) represents a key component in the regulation of aliphatic methionine-derived GSL biosynthesis in *A. thaliana.* In addition, Hirai *et al.* [16] described that MYB29 probably plays an important role for methyl jasmonate-mediated induction of a set of aliphatic GSL biosynthetic genes in *A. thaliana.* The transcription of *MYB29* in the stems was 46-, 11-, and 92-fold higher, respectively, than that of flowers, young leaves, and old leaves.

Additionally, the transcript level of *MYB28-2* in the stems was also 552-, 184-, and 16-fold higher, respectively, than that of seeds, roots, and young leaves, whereas the transcript level of *MYB28-1* in the flowers was 44-, 4.8- and 1.5-fold higher than that of seeds, old leaves, and stems. The highest *MYB28* expression level was detected in inflorescences of flowering plants and in old leaves, the sites of accumulation of aliphatic GSLs in *Arabidopsis* [14]. Gigolashvili *et al.* [13] identified that HIG1 (high indolic glucosinolate)/MYB51 regulates indolic GSL biosynthesis in *A. thaliana* and plays a role in biotic stress responses. *Arabidopsis HIG1/MYB51* was expressed in roots but not in mature flowers or siliques and *MYB51* promoter activity was highest in the vegetative parts of the plants, mainly in mature rosette leaves [13]. In contrast to *Arabidopsis*, among *B. rapa MYB51* isoforms, *MYB51-2* was expressed only in seeds, while *MYB51-1* and *MYB51-3* were expressed in other organs, except for the flowers and stems. In the case of *MYB122, MYB122-2* transcription was highest in young leaves, whereas among the *MYB34* isoforms, *MYB34-4* transcript level in the roots was 9, 4.5 and 3 times higher compared with seeds, old leaves, and young leaves. Transcription of *MYB34-2* and *MYB34-3* was only observed in seeds, young leaves, and roots, whereas *MYB34-1* was expressed in all organs. 

In *Arabidopsis*, *MYB34* overexpressing plants exhibited defectiveness in the development of generative organs, the root system, as well as the vegetative biomass [13]. Jin *et al.* [26] reported that MYB51 and MYB122 could act as activators or repressors, depending on the context, in a similar way like AtMYB4. The expression of GSL biosynthetic genes, that is, *CYP79B2*, *UGT74B1*, *CYP79F1*, *CYP79F2*, *IQD1*, and *Dof1.1* in *A. thaliana* often exhibited to be restricted to vascular tissues [10,12,25,27,28]. In spite of the common precursors between IAA and IG biosynthetic pathways, they could be specifically regulated by the different activities of MYB122, MYB34, and MYB51 [13].

*Dof1.1-1* and *Dof1.1-2* expression showed similar patterns in all organs, except for seeds. *IQD1-1*, which is involved in both indolic and aliphatic GSLs biosynthesis, showed the highest expression level in young and old leaves. The expression of *IQD1-1* in the young leaves was 19-, 13.6-, and 4.2-fold higher, respectively, than in seeds, roots, and stems, whereas *IQD1-1-2* transcript level in roots was expressed 29- and 2.9-fold higher, respectively, than in the stems and seeds. Zang *et al.* [29] described that the expression of *B. rapa* desulfoglucosinolatesulfotransferases (*BrST*) isoforms involved in core GSLs biosynthesis in mature leaf and root was higher than other tissues, displaying functional redundancy for differential expression. In this study, *MYB28*, *MYB29,* and *Dof1.1* isoforms, involved in aliphatic GSLs biosynthesis, exhibited high expression level in the flowers, stems, and seeds, whereas indolic GSLs biosynthesis genes showed high level in a variety of organs. Therefore, we suggest that expression of MYB TFs isoforms involved in GSLs could differ tremendously depending on tissue type in Chinese cabbage.

### 2.3. GSLs Analysis of Different Organs

Individual GSLs were separated by HPLC and identified based on retention time of HPLC and our GSL database. Moreover, the GSLs were confirmed by LC-ESI-MS in positive mode (Table 2). Organs including flowers, stems, young leaves, old leaves, roots, and seeds of Chinese cabbage were quantified with an external standard, sinigrin, and response factor (Table 3) [2, ISO 1992]. Five aliphatic GSLs (progoitrin, sinigrin, glucoalyssin, gluconapin, and glucobrassicanapin), 2 indolic GSLs (glucobrassicin and 4-methoxyglucobrassicin), and 1 aromatic GSL (gluconasturtiin) were detected in all organs. Gluconapin and sinigrin, from the aliphatic GSLs, showed the highest levels in the seeds (60.13 and 3.58 μmol/g DW, respectively), whereas old leaves had the lowest levels (0.34 and 0.11 μmol/g DW, respectively). This result was consistent with gene expression level of *Dof1.1* involving aliphatic GSL biosynthesis. Very recently, Cartea *et al.* [30] demonstrated that total GSL content ranged from 19 to 37.3 μmol/g DW in early and extra-late groups, respectively, and from 19.5 to 36.3 μmol/g DW for turnips and turnip greens groups, respectively in leaves of *B. rapa.*

**Table 2:** Glucosinolates identified from Chinese cabbage by LC-ESI-MS.

Trivial name *	Systematic names	Compound groups	[M+H]^+^ *m/z*	Response factor [2, ISO 1992]
Progoitrin	4-Methylsulfinylbutyl GSL	Aliphatic	310	1.09
Sinigrin	2-Propenyl GSL	Aliphatic	280	1.00
Glucoalyssin	5-Methylsufinylpentyl GSL	Aliphatic	372	1.07
Gluconapin	3-Butenyl GSL	Aliphatic	294	1.11
Glucobrassicanapin	Pent-4-enyl GSL	Aliphatic	308	1.15
Unknown 1	Unknown	Unknown	327	1.00
Unknown 2	Unknown	Unknown	292	1.00
Glucobrassicin	3-Indolymethyl GSL	Indolic	369	0.29
4-Methoxyglucobrassicin	4-Methoxy-3-indolylmethyl GSL	Indolic	399	0.25
Neoglucobrasscin	N-Methoxy-3-indolylmethyl GSL	Indolic	399	0.20
Gluconasturtiin	2-Phenethyl GSL	Aromatic	344	0.95

* The elution order of HPLC chromatogram (data not shown) was as follow: progoitrin-sinigrin-glucoalyssin-unknown 1-gluconapin-unknown2-glucobrassicanapin-glucobrassicin-4-methoxyglucobrassicin-gluconasturtiin-neoglucobrasscin.

Gluconapin was the major glucosinolate in 113 varieties of turnip greens (*B. rapa*) from northwestern Spain grown at two sites [30]. Progoitrin and glucobrassicanapin were detected mainly in the stems, young leaves, old leaves, and flowers. Padilla *et al.* [31] demonstrated that glucoraphanin presenting in 27 of 113 varieties of turnip greens (*B. rapa*) should be studied more exhaustively since this aliphatic glucosinolate is the precursor of sulforaphane, a potent anti-cancer isothiocyanate. In this study, progoitrin exhibited 18.2% of the total GSLs content. Kim *et al.* [32] reported that glucobrassicanapin has the greatest proportion (21.1%) of total GSLs in the leaves. However, the glucobrassicanapin content was found to be highest in the flowers in this study. 

From the aromatic GSLs, the level of gluconasturtiin in the roots (8.42 μmol/g DW) was 4–15 fold higher than that in the other organs. The total GSL content according to the site (ranked in descending order) is as follows: seeds > flowers > young leaves > stems > roots > old leaves. Aliphatic GSLs showed 63.3% of the total GSLs content, followed by aromatic GSL (19.0%), indolic GSLs (10%), and unknown GSLs (7.7%) in different organs of Chinese cabbage. In addition, total aliphatic, indolic, aromatic, and unknown GSLs content showed 65.7, 11.7, 12.7, and 9.7%, respectively in leaves. Cartea *et al.* [30] reported that aliphatic GSLs were predominantly exhibited 95.5% of the total GSLs content, followed by indolyl GSLs (2.4%) and aromatic GSL (2.1%) in leaves of vegetable turnip rape (*B. rapa* L. var. *rapa*).

**Table 3:** Glucosinolate content (µmol/g DW) in different organs of Chinese cabbage.

Trivial name	Flower	Stem	Young leaf	Old leaf	Root	Seed
Progoitrin	5.53 ± 0.04	3.78 ± 0.02	4.03 ± 0.10	1.11 ± 0.05	1.11 ± 0.03	3.26 ± 0.21
Sinigrin	2.23 ± 0.22	1.17 ± 0.02	0.34 ± 0.03	0.11 ± 0.03	0.12 ± 0.00	3.58 ± 0.79
Glucoalyssin	0.68 ± 0.22	0.18 ± 0.12	0.22 ± 0.10	0.19 ± 0.01	0.11 ± 0.00	0.47 ± 0.21
Gluconapin	4.51 ± 0.25	1.35 ± 0.10	1.57 ± 0.19	0.34 ± 0.03	0.42 ± 0.02	60.13 ± 1.76
Glucobrassicanapin	10.85 ± 0.20	4.61 ± 0.18	4.32 ± 0.16	0.89 ± 0.04	1.73 ± 0.04	4.92 ± 0.90
Unknown 1	1.08 ± 0.11	0.99 ± 0.08	0.99 ± 0.10	0.29 ± 0.04	0.62 ± 0.07	0.41 ± 0.08
Unknown 2	0.52 ± 0.23	0.47 ± 0.24	0.50 ± 0.22	0.16 ± 0.00	ND	7.34 ± 1.34
Glucobrassicin	1.96 ± 0.44	0.92 ± 0.14	0.43 ± 0.05	0.37 ± 0.20	0.72 ± 0.05	0.90 ± 0.09
4-Methoxyglucobrassicin	0.20 ± 0.03	0.41 ± 0.05	1.14 ± 0.06	0.15 ± 0.04	0.19 ± 0.02	1.27 ± 0.03
Neoglucobrassicin	0.14 ± 0.02	0.08 ± 0.04	0.14 ± 0.04	0.09 ± 0.04	0.30 ± 0.00	0.12 ± 0.02
Gluconasturtiin	2.10 ± 0.13	0.80 ± 0.09	1.89 ± 0.16	0.65 ± 0.23	8.42 ± 0.16	0.91 ± 0.12
Total	29.81 ± 0.69	14.77 ± 0.53	15.59 ± 0.42	4.30 ± 0.38	13.66 ± 0.23	83.38 ± 2.98

* ND- not detected. Values are the means from three independent experiments ± SD (n = 3). * GSLs were analyzed by slightly modifying the protocol used in a previous study [14,15].

In our study, progoitrin and glucobrassicanapin, which belongs to the aliphatic GSLs group, were highly detected in flowers (Table 3). The transcript level of *MYB28-1* in flowers was higher compared to other organs (Figure 3). It seems that there is a relationship between aliphatic GSLs content and gene expression level of *MYB28-1*. The rank of total aliphatic GSLs content was as follows: seeds (72.36 μmol/g DW) > flowers (23.80 μmol/g DW) > stems (11.09 μmol/g DW) > young leaves (10.48 μmol/g DW) > roots (3.49 μmol/g DW)> old leaves (2.64 μmol/g DW). On the other hand, the rank of total indolic GSLs content was flowers (2.30 μmol/g DW) > seeds (2.29 μmol/g DW) > young leaves (1.71 μmol/g DW) > stems (1.41 μmol/g DW) > roots (1.21 μmol/g DW) > old leaves (0.61 μmol/g DW). In this study, the transcript levels of *MYB34*, *MYB51*, and *MYB122* were higher in the seeds and roots compared to other organs. Furthermore, the two indolic GSLs, glucobrassicin and 4-methoxyglucobrassicin, were also highly accumulated in the flowers and seeds. These results reveal that there is somewhat a correlation between the expression levels of indolic GLSs responsive genes and their related compounds. The reason of having relationship between gene expression and GSL content is somewhat, might be due to presence of many genes relating in amino acid chain elongation, core structure formation, and secondary modification taking part in GSL biosynthesis as well as MYB transcription factors.

## 3. Experimental

### 3.1. Plant Materials and Growth Conditions

Seeds of Chinese cabbage (*B. rapa* ssp. *Pekinensis* cv. Asia Alpine), were purchased from Asia Seed Co., Ltd (Seoul, Korea). Chinese cabbages were grown at the experimental farm of Chungnam National University (Daejeon, Korea). After 4 months, different organs (seeds, flowers, stems, young leaves, old leaves, and roots) were excised from several plants. All samples were immediately frozen in liquid nitrogen and then stored at −80 °C and/or freeze-dried for RNA isolation and/or high-performance liquid chromatography (HPLC) analysis.

### 3.2. Bioinformatic Analysis

For sequence data of *B. rapa* ssp. *pekinensis*, Brassica genome database (http://brassicadb.org/brad/glucoGene.php) [21] was utilized. In MYB29 isoform, Bra009245 (MYB29-1) is almost similar to Bra005949 (MYB29-2). Thus, Bra009245 gene was not used in this study. For protein subcellular localization prediction, TargetP (http://www.cbs.dtu.dk/services/TargetP/) [33], ChloroP (http://www.cbs.dtu.dk/services/ChloroP/) [33], and Wolf PSORT (http://wolfpsort.org/) [34] were used. The phylogenetic relationships of MYB TFs were analyzed with the TreeTop Phylogenetic tree prediction program (http://www.genebee.msu.su/services/phtree_reduced.html) [35] by applying the cluster algorithm. In the bootstrap, a multiple Sequence Alignment was resampled 100 times. 

### 3.3. cDNA Synthesis and Quantitative Real-time PCR Analysis

Total RNA was isolated from different organs using Total RNA extraction Kit (Geneaid, New Taipei, Taiwan). For qRT-PCR, first-strand cDNA was synthesized from the total RNA with ReverTra Ace-α-(Toyobo, Osaka, Japan) Kit and oligo (dT)_20_ primer. The protocol for the reverse transcriptase polymerase chain reaction (RT-PCR) is as follows: Eleven micro liters of RNase-free water was mixed with 1 µg of total RNA (1 µL), 2 µL of 10° buffer, 1 µL of 10 mM each dNTP, 2 µL of 10 µM oligo dT primer, 1 µL of 40 µ/µL RNase inhibitor, and 1 µL of 4 u/µL reverse transcriptase to a final volume of 20 µl. The mixture was reamplified for 20 min at 42 °C and heated for 5 min at 99 °C. In order to design the primers for real time PCR, we performed DNA sequence alignment for each MYB transcription factor in Chinese cabbage and gene-specific primers were designed using an online program (http://web.bioneer.co.kr/tools/tmcalculator.jsp) [36] (Table S1). The SYBR Green qRT-PCR assay was carried out in a total volume of 20 µL, containing 10 µL of 2X SYBR Green Real time PCR master mix (Toyobo, Osaka, Japan), 0.5 µM (each) of specific primers, and template cDNA was diluted 10-fold. The amplification program consisted of one cycle of 95 °C for 3 min, followed by 40 cycles of 95 °C for 15 s, 72 °C for 20 s and annealing temperature of each gene was shown (Table S1). The reaction was performed in triplicate on a Mini Opticon Real-Time PCR System (Bio-Rad, Hercules, CA, USA) with the SYBR Green Real-time PCR Master Mix (Toyobo, Osaka, Japan). The *actin* gene (GenBank accession No. FJ969844) was selected as a reference gene because actin gene exhibited expression stability in different organs. The actin gene has also been used as a reference gene in several studies [37,38,39].

### 3.4. Chemicals

(−)-Sinigrin (2-propenyl glucosinolate) hydrate from horseradish for use as an external standard, and aryl sulfatase (Type H-1, EC 3.1.6.1) for desulfation of glucosinolates were purchased from Sigma–Aldrich (St. Louis, MO, USA). DEAE-Sephadex A-25 for loading into a mini-column (1,000 µL-Pasteur pipette) was provided by Amersham Biosciences (Uppsala, Sweden). HPLC-grade acetonitrile (CH_3_CN) and methanol (MeOH) were supplied by J. T. Baker (Phillipsburg, NJ, USA). Ultrapure water having a resistivity of 18.2 MΩ/cm was produced by a PureLab Option from ELGA Labwater (Model LA 621, Marlow, UK).

### 3.5. Extraction of Desulfo-glucosinolates (DS-GSLs) and HPLC Analysis

DS-GSLs were extracted using a slight modification of the procedures reported in a previous study [40,41]. Briefly, crude GSLs from 100 mg of freeze-dried powder were extracted with 1.5 of boiling 70% (v/v) MeOH at 70 °C for 5 min at a water-bath. After centrifugation at 12,000 rpm at 4 °C for 10 min, the supernatant was collected into a 5 mL test tube, and the residue was re-extracted twice as described above. The combined supernatants were taken as the crude GSL extracts. The extracts were loaded into a mini-column previously packed with DEAE-Sephadex A-25 and desulfated by the addition of 75 μL of an aryl sulfatase solution. DS-GSLs samples were eluted into a 2 mL microcentrifuge tube with 0.5 mL (×3) of ultrapure water.

The separation of DS-GSLs was carried out on a reversed-phase Inertsil ODS-3 column (150 × 3.0 mm i.d., particle size 3 μm; GL Sciences, Tokyo, Japan) with an E type cartridge guard column (10 × 2.0 mm i.d., 5 μm) using an Agilent Technologies 1,200 series HPLC system (Palo Alto, CA, USA). The detection wavelength, column oven temperature and flow rate were set at 227 nm, 40 °C and 0.2 mL/min, respectively. The mobile phase consisted of ultrapure water (solvent A) and CH_3_CN (solvent B). The gradient programs were as follows: A linear step from 7% to 24% of solvent B for 18 min, 24% of solvent B for the next 14 min, then kept constant at solvent B 24% for 3 min followed by a rapid drop to 7% solvent B at 32.1 min, and kept constant at solvent B 7% for 8 min (total 40 min). The individual GSLs were identified based on their HPLC retention times and our data base and quantified with the external standard, sinigrin (0.5 mg/5 mL for its desulfation, but it was diluted 4-times before HPLC injection) passed through the same extraction process together with sample preparation, with their HPLC area and response factor (ISO 9167-1, 1992).

### 3.6. LC/ESI-MS Analysis for Quantitation of Desulfoglucosinolates (DS-GSLs)

The MS data were acquired by electrospray ionization (ESI)- mass spectrometry with an API 4000 Q TRAP system (Applied Biosystems, Foster City, CA, USA) in positive ion mode ([M+H]^+^) that was equipped with an Agilent 1,200 series HPLC system. The MS operating conditions were as follows: scan range, *m/z* 100–800 (scan time, 1.0 s); curtain gas (20 psi), nebulizing gas (50 psi), heating gas (50 psi) by high purity nitrogen (N_2_); heating gas temperature, 550 °C; ion spray voltage, 5,500 V; declustering potential, 100 V; entrance potential, 10 V.

### 3.7. Statistical Analysis

The data were analyzed by using the computer software Statistical Analysis System (SAS version 9.2, SAS Institute Inc., Cary, NC, USA, 2009). All data are given as the mean and standard deviation of triplicate experiments. Treatment mean comparisons were performed with the Least Significant Difference (LSD).

## 4. Conclusion

In this study, we performed qRT-PCR of TFs factors involved GSLs biosynthesis and analyzed GSLs content in different organs of Chinese cabbage. Even though expression levels of *MYB28* and *MYB29* in seeds was not high, transcript levels of flowers and stems were higher compared with other organs. Our results showed distinctly that MYB28-1 regulate aliphatic GSLs biosynthesis in Chinese cabbage, whereas MYB34, MYB51, and MYB122 involving in indolic GSLs biosynthesis could differ depending on different organ in Chinese cabbage. Especially, there is a correlation between aliphatic GSLs content and gene expression level of *MYB28-1*, *Dof1.1-1*, and *Dof1.1-2.* Therefore, our results may be helpful in understanding the mechanism of MYB TFs regulating GSL biosynthesis in Chinese cabbage.

## Supplementary Materials

Supplementary materials can be accessed at: http://www.mdpi.com/1420-3049/18/7/8682/s1.
