# Supplementary material for: MYB Transcription Factors Regulate Glucosinolate Biosynthesis in Different Organs of Chinese Cabbage (Brassica rapa ssp. pekinensis)
Source: Molecules. 2013 Jul 22;18(7):8682–95. doi: 10.3390/molecules18078682 (PMC6269701; doi:10.3390/molecules18078682)
Supplement: Supplementary file 1 [file molecules-18-08682-s001.pdf]

Supplementary Information

Table S1. List of real-time RT-PCR primers used in this study.

Primer	Sequence (5' → 3')	Annealing Temp (°C)	Size (bp)
Bra031588-F (*Dof1.1-1*)	AACAGTCATGACAACAACAACAGTGA	52	197
Bra031588-R	GGTTGTTCTTCCATCTTCACCG		
Bra030696-F (*Dof1.1-2*)	ATGGACGAAACATAGCAGCTCC	52	248
Bra030696-R	TCTAGACAAGTTAACCAAACCGGGT		
Bra034081-F (*IQD1-1-1*)	AGAGTTGATAAGGTCAAACGTAAGAAG	50	218
Bra034081-R	CTTCTTGCCAAATGTCCTCTAAATA		
Bra001299-F (*IQD1-1-2*)	CCAAGAGCTCTGATAAGAACCGTAG	51	273
Bra001299-R	TGGTCGCAGCAGCTGAGC		
Bra012961-F (*MYB28-1*)	ACCATACTGTCAACACGCCTCC	52	219
Bra012961-R	CAGAAGTGACCTTAGCCGCAAC		
Bra035929-F (*MYB28-2*)	ACCCTACAGTACCTGAGAATTTGCA	51	200
Bra035929-R	ACTTGATTTCTTAAAACGTTTCTTGC		
Bra029311-F (*MYB28-3*)	AATACCATATGAAAGCTTTTCCAATG	52	238
Bra029311-R	AGGAAGATCATGACCATACTGATCG		
Bra005949-F (*MYB29*)	CTGTCTCCTCCGTGTCTCAA	54	148
Bra005949-R	CCTCGGCTGCATTGTTACTA		
Bra013000-F (*MYB34-1*)	ACACCGGCGACGTCGATTC	53	229
Bra013000-R	TCTAACTCCTCCATAAGGCCAACA		
Bra035954-F (*MYB34-2*)	CACAATCGTCGCCGACG	51	270
Bra035954-R	GGCGAACCCTTAATCTCTCCG		
Bra029350-F (*MYB34-3*)	AACAAAGCGGCGGCAAG	52	186
Bra029350-R	ATCCCTCAACTCTTCCATAATGCAA		
Bra029349-F (*MYB34-4*)	CCAACCGGTTTCGAACCAG	52	264
Bra029349-R	GAATACGTCAAGATCATCGGAGAA		
Bra025666-F (*MYB51-1*)	GATCTCCGAAACCAGCAAATCA	51	240
Bra025666-R	GTAGTAATGAGTGGGCCACCACT		
Bra031035-F (*MYB51-2*)	ATCTCCGACAAATCAGAAAACCTC	51	236
Bra031035-R	GTAGTAGTAAGTGGGCCACCACTTC		
Bra016553-F (*MYB51-3*)	TCAGAAAATTTGCAGGATATTCTGG	51	230
Bra016553-R	ACGGTGACACTTGTAGTAATAGTAGTAGTGTTA		
Bra015939-F (*MYB122-1*)	TGTTTCTCATGATGATGAAGATTTCTTG	53	184
Bra015939-R	CCAGTTGTCAATCCCTTCAAAGG		
Bra008131-F (*MYB122-2*)	TTGAATGATGTTGTATCTCATGATGATG	53	190
Bra008131-R	AGTTGTCAATCCCTTCAAAGGAAACA		
BraActin-F	TAGTGTTGTTGGTAGGCCAAGACAT	53	188
BraActin-R	GGAGCTCGTTGTAGAAAGTGTGATG		
